# Population pharmacokinetics and pharmacodynamics of the artesunate–mefloquine fixed dose combination for the treatment of uncomplicated falciparum malaria in African children

**DOI:** 10.1186/s12936-019-2754-6

**Published:** 2019-04-18

**Authors:** Monia Guidi, Thomas Mercier, Manel Aouri, Laurent A. Decosterd, Chantal Csajka, Bernhards Ogutu, Gwénaëlle Carn, Jean-René Kiechel

**Affiliations:** 1 School of Pharmaceutical Sciences, University of Geneva, University of Lausanne, Geneva, Switzerland; 2 Laboratory and Service of Clinical Pharmacology, Centre Hospitalier Universitaire Vaudois and University of Lausanne, Lausanne, Switzerland; 3 Kenya Medical Research Institute, Kisumu, Kenya; 4 Drugs for Neglected Diseases initiative, Geneva, Switzerland

**Keywords:** Population pharmacokinetics, Mefloquine, Artesunate, Dihydroartemisinin

## Abstract

**Background:**

The World Health Organization (WHO) recommends combinations of an artemisinin derivative plus an anti-malarial drug of longer half-life as treatment options for uncomplicated *Plasmodium falciparum* infections. In Africa, artesunate–mefloquine (ASMQ) is an infrequently used artemisinin-based combination therapy (ACT) because of perceived poor tolerance to mefloquine. However, the WHO has recommended reconsideration of the use of ASMQ in Africa. In this large clinical study, the pharmacokinetics (PK) of a fixed dose combination of ASMQ was investigated in an African paediatric population to support dosing recommendations used in Southeast Asia and South America.

**Methods:**

Among the 472 paediatric patients aged 6–59 months from six African centres included in the large clinical trial, a subset of 50 Kenyan children underwent intensive sampling to develop AS, its metabolite dihydroartemisinin (DHA) and MQ PK models. The final MQ PK model was validated using sparse data collected in the remaining participants (NONMEM^®^). The doses were one or two tablets containing 25/55 mg AS/MQ administered once a day for 3 days according to patients’ age. A sensitive LC–MS/MS method was used to quantify AS, DHA and MQ concentrations in plasma. An attempt was made to investigate the relationship between the absence/presence of malaria recrudescence and MQ area under the curve (AUC) using logistic regression.

**Results:**

AS/DHA concentration–time profiles were best described using a one-compartment model for both compounds with irreversible AS conversion into DHA. AS/DHA PK were characterized by a significant degree of variability. Body weight affected DHA PK parameters. MQ PK was characterized by a two-compartment model and a large degree of variability. Allometric scaling of MQ clearances and volumes of distribution was used to depict the relationship between MQ PK and body weight. No association was found between the model predicted AUC and appearance of recrudescence.

**Conclusions:**

The population pharmacokinetic models developed for both AS/DHA and MQ showed a large variability in drug exposure in the investigated African paediatric population. The largest contributor to this variability was body weight, which is accommodated for by the ASMQ fixed dose combination (FDC) dosing recommendation. Besides body weight considerations, there is no indication that the dosage should be modified in children with malaria compared to adults.

*Trial registration* Pan African Clinical Trials Registry PACTR201202000278282 registration date 2011/02/16

**Electronic supplementary material:**

The online version of this article (10.1186/s12936-019-2754-6) contains supplementary material, which is available to authorized users.

## Background

The World Health Organization (WHO) estimates a significant 18% reduction in the incidence of malaria along with a considerable 28% decrease in the malaria mortality rate between 2010 and 2017 [1]. Despite this substantial progress, the disease still caused an estimated 435,000 deaths worldwide, mostly in Africa (93%) and in children under 5 years of age (61%) [1]. Artemisinin-based combination therapy (ACT) is the first-line treatment for uncomplicated *Plasmodium falciparum* infection, the predominant cause of malaria in Africa, recommended by the WHO since 2001 [2]. These combinations involve a rapidly eliminated and fast-acting artemisinin derivative together with a much more slowly eliminated drug that kills the remaining parasites. One of the five WHO recommended artemisinin-based combinations is artesunate (AS) associated with mefloquine (MQ), extensively used in Asia and Latin America for the last 20 years [3]. This combination is less commonly selected in Africa, because of the availability of other affordable and already registered artemisinin-based combinations [4], as well as existing concerns about MQ tolerability [5, 6]. However, the WHO has recommended reconsideration of the use of ASMQ in Africa in order to increase the number of artemisinin-based combinations available, with the consequent reduction of the risk of developing drug resistance [4].

The development of a fixed-dose combination (FDC) of AS and MQ was begun in 2002 by the Drugs for Neglected Diseases *initiative* (DND*i*) with the fixed-dose artesunate-based combination therapy (FACT) Consortium [3]. This combination has been demonstrated to be efficacious and safe in Asia and Latin America [7–9], but there is still limited experience with its use in Africa. Therefore, an open-label, prospective, randomized, controlled, multi-centre, non-inferiority clinical trial evaluating the efficacy, safety and pharmacokinetics of the ASMQ FDC versus artemether–lumefantrine (AMLF) in children aged 6–59 months was conducted in Africa by DND*i* (Pan African Clinical Trials Registry number PACTR201202000278282). Because MQ dose splitting into three equal daily doses has been shown to optimize treatment compliance and to improve MQ tolerability [10, 11], FDC ASMQ dispersible tablets were administered over three consecutive days based on the patients’ age. The efficacy of ASMQ was found to be non-inferior to the efficacy of AMLF and the safety of the two treatments was found to be similar with low risk of repeated early vomiting, indicating that ASMQ is a valuable treatment option for children younger than 5 years with uncomplicated falciparum malaria in Africa [12]. Within the framework of this previous study, a pharmacokinetic study was conducted to characterize ASMQ FDC pharmacokinetics in the African paediatric patient population, to compare it to data gathered in adult patients and volunteers, to validate the recommended treatment regimen, and to explore the relationships between drug exposure and treatment outcomes.

## Methods

### Study design and participants

The clinical trial was carried out in six African centres: three in Tanzania, two in Burkina Faso and one in Kenya. Written informed consent from a parent/guardian was required to enrol children younger than 5 years in the trial, who were infected by *P. falciparum*, as confirmed by microscopy (density between 2000 and 200,000 asexual parasites/µL), and with fever equal to or higher than 37.5 °C. Exclusion criteria were children with body weight less than 5 kg, signs of severe/complicated malaria, febrile conditions caused by diseases other than malaria, a known hypersensitivity to the study drugs, a mixed plasmodium infection, a history of anti-malarial treatment in the 2 weeks preceding the trial or 4 weeks in case of mefloquine and piperaquine, prior participation in a therapeutic trial within 3 months or inability to tolerate oral medication. Patients were followed up to day 63 after start of treatment or to the first recurrence of infection. The study protocol was reviewed and approved by national and independent ethics committees of all participating centres.

Of the 945 patients enrolled in the trial, 473 were randomized to the ASMQ arm (one of them was never dosed) and 472 were randomized to the AMLF arm. The pharmacokinetic analysis described here was performed on the 472 patients who received ASMQ.

Administered doses for these patients were one or two dispersible tablets containing 25 mg AS and 55 mg MQ once a day for three consecutive days to children aged from 6 to 11 months and from 12 to 59 months, respectively. Clinical and parasitological examinations were scheduled at baseline, i.e. before drug administration, at day 0 (D0), D1, D2, D3, D7, D14, D21, D28, D35, D42, D49, D56 and D63 and on any other day if the patient spontaneously returned and parasitological reassessment was required (as per protocol). A margin of ± 2 days to the assigned day of visit was allowed from D7 onward. In case of recurrence of parasitaemia on D7, D14, D21, D28, D35, D42, D49, and D56 the date was recorded and the type of recurrence was determined by PCR (appearance of new infection, malaria recrudescence, missing PCR information or undetermined type).

According to the study protocol, the first fifty children from Kenya enrolled in the ASMQ arm underwent intensive blood sampling: at baseline, on D0 after drug administration (until 6 h after first dosing), D2 (until 6 h after the third dose), D3 (72 h after first dose), D7 and on one other occasion on day 28, 35, 42, 49, 56 or 63. Two blood samples, at baseline and on D7, were collected for all the other participants. Additionally, for all patients with recurrence of parasitaemia, a blood sample was taken on the day of failure.

### Analytical methods

The mass spectrometry assay for AS, DHA and MQ used for the analysis of study samples is an adaptation of a previously published validated multiplex method [13]. The assay has been further improved by the use of stable isotopically labelled internal standards for MQ (mefloquine-d9) and DHA (DHA-13Cd4) to circumvent the potential matrix effect that may affect the accuracy of mass detection.

The mobile phase was delivered at a flow rate of 0.3 mL/min on a 2.1 mm × 75 mm XSelect HSS 3.5 μm column (Waters, Milford, MA, USA), using solvent A (2 mM ammonium acetate + 0.1% FA) and solvent B (MeCN + 0.1% FA) distributed according to the following stepwise gradient program: 98% A: 0 min; 98% A → 15% A: from 0.0 min → 13.0 min followed by a re-equilibration step to the initial solvent proportions. The retention time of mefloquine/mefloquine-d9, DHA/DHA-13Cd4 and artesunate is 7.4 min, 8.2 min and 9.2 min, respectively. The chromatographic system was coupled to a triple stage quadrupole (TSQ) Quantum Ion mass spectrometer (MS) from Thermo Fischer Scientific (Waltham, MA, USA) equipped with an Ion Max electrospray ionization (ESI) interface. The limits of quantification (LOQ) of the method are 2.5 ng/mL for MQ and 2 ng/mL for AS and DHA.

Plasma samples were isolated by centrifugation and stored at − 20 °C until batch analysis. Briefly, 100 μL of plasma sample were mixed with 50 µL internal standard (DHA-13Cd4 at 130 ng/mL; mefloquine-d9 at 43 ng/mL) and extracted with 600 µL of acetonitrile. The supernatant (700 µL) was evaporated under nitrogen at room temperature and was reconstituted in 150 µL of MeOH/ammonium acetate 2 mM (1:1) adjusted with formic acid at 0.1%, vortex-mixed and centrifuged again. The samples were maintained at +5 °C in autosampler racks throughout the analytical series. The injection volume was 20 μL.

The method is precise (with mean inter-day CV % < 10%), and accurate (inter-day deviation from nominal values < 5%). Since its initiation, the laboratory has participated in the Pharmacology Proficiency Testing Programme for anti-malarial drugs (http://www.wwarn.org/toolkit/qaqc) organized by the World Wide Antimalarial Resistance Network WWARN (http://www.wwarn.org/).

#### Pharmacokinetics analysis

Non-linear mixed effects modelling program (NONMEM^®^, version 7.3) [14] with the Perl-Speaks NONMEM^®^ (PsN) toolkit (version 3.7.6) [15] was used to estimate average population pharmacokinetic parameters and their associated between-subject variability (BSV) and to identify factors that influence them. MQ and AS/DHA pharmacokinetic models were developed on the data collected from 50 Kenyan patient subjects with extensive sampling. Molar units were used for AS/DHA pharmacokinetic analyses. Because of the very fast rate of AS and DHA elimination and the selection of the trial sampling times, an external model validation could only be performed for MQ on the clinical trial data not used for model-building. Graphical exploration and statistical analyses were performed by means of the R package (version 2.15.1, R Development Core Team, http://www.r-project.org/).

### Structural and statistical model

A stepwise modelling approach was undertaken to identify models that best described the MQ and AS/DHA pharmacokinetics. Multi-compartment dispositions with first-order absorption and elimination processes were compared for MQ. Due to the restricted amount of AS and DHA data, drug and metabolite pharmacokinetics were modelled simultaneously and directly described by means of a one compartment model with linear absorption and elimination. Moreover, since AS is rapidly and almost completely hydrolysed in DHA, its elimination was assumed to occur exclusively via irreversible conversion to DHA [16, 17]. An adequate AS absorption rate constant (K_a_) estimation could not be made because of the small number of samples collected right after dose intake (one sample at maximum for each enrolled child on the first and third treatment day). K_a_ was thus fixed to 3.2 h^−1^, the mean of previously published estimates retrieved from papers using a first-order process to depict AS absorption [17, 18].

Parameterization was performed in terms of clearances (CL for drugs and CL_M_ for metabolite), inter-compartmental clearance (Q), central (V_C_ for drugs and V_M_ for metabolite) and peripheral (V_P_) volumes of distribution and K_a_. The metabolic conversion rate from AS to DHA was estimated by CL/V_c_ as previously discussed. AS and MQ relative bioavailability (F1, fixed to 100% and with estimated BSV) were also tested for AS/DHA and MQ to account for dose variation with respect to the nominal value due to the administration of water dispersible tablets. Since the ASMQ combination is administered orally, the pharmacokinetic parameter estimates represent apparent values.

Exponential errors were assumed to capture BSV in all the pharmacokinetic parameters. Proportional, additive and combined proportional-additive error models were compared to describe drugs and metabolite intra-patient (residual) variability. Finally, the correlation between AS and DHA concentration measurements was tested using the L2 function in NONMEM^®^.

### Covariate analysis

Available covariates were: body weight (BW), height/length, age, sex, creatinine, total bilirubin (BIL), aspartate (AST) and alanine (ALT) aminotransferases, haemoglobin (Hb), haematocrit (Ht), total parasitaemia and co-medications categorized as CYP3A4 inducers. Visual inspection of the correlation between post hoc individual estimates of the pharmacokinetic parameters and the available patients’ characteristics was initially conducted to identify potential physiologically plausible relationships. Creatinine clearance was not evaluated since MQ elimination occurs mainly through non-renal processes and AS is completely converted into DHA, which is eliminated via glucuronidation [16]. A stepwise forward insertion/backward deletion approach was then undertaken. Potential covariates influencing the kinetic parameters were first incorporated one at a time and tested for significance (univariate analysis). Sequential multivariate combinations of the identified factors were investigated to discard redundancies and to build an intermediate model with all the most important covariates (multivariate analysis). Finally, backward deletion consisted of removing covariates one at a time from the intermediate model, starting from the most insignificant until no further deterioration of the model was observed.

The influence of body weight on all MQ and DHA pharmacokinetic parameters (PAR) was tested using allometric scaling:

1 $$ PAR = \theta *\left( {\frac{BW}{MBW}} \right)^{PWR} $$

with θ PAR population estimate, MBW the median population body weight and PWR the function power fixed to 0.75 for clearances and 1 for volumes of distribution [19]. A linear relationship between the typical value of a parameter and all the other covariates (continuous centered on the population median; dichotomous coded as 0 and 1) was used. Additionally, AST, ALT and BIL were implemented in the model as dichotomous variables, by introducing a boundary condition, i.e. below or exceeding 1.5 times the upper limit of normal (ULN). Children’s age was used to investigate the impact of organ maturation on MQ and DHA clearances, using the following equations, in addition to the simple linear one:

2 $$ CL = \theta *\frac{1}{{1 + \left( {\frac{AGE}{{TM_{50} }}} \right)^{ - Hill} }} $$

3 $$ CL = \theta *\left( {MAT_{mag} + \left( {1 - MAT_{mag} } \right)*\left( {1 - e^{{ - AGE*K_{mat} }} } \right)} \right) $$

where Hill is the sigmoid power, TM_50_ the AGE at 50% of maturation, MAT_mag_, the maturation magnitude for age, and K_mat_ the age maturation rate constant [20, 21]. The population median covariate value was assigned to patients with missing information.

The acute phase of malaria is associated with altered gastrointestinal motility and an increased likelihood of vomiting. In the three-daily dose ASMQ regimen, the second dose is administered when the patient is in an improved state of health, thanks to the first dose of AS, that kills most of the parasites [22]. The potential impact of parasitaemia on AS and MQ F1 was studied using a linear model of log-transformed (base 10) parasite counts measured at baseline of each ASMQ administration day. Missing parasitaemia information was imputed at the median value of the specific study day. Treatment day (0 vs. 1 and 2), considered as a surrogate marker of the rapid improvement in health due to the first AS dose, was also evaluated on AS and MQ F1. Since parasite counts and treatment day are correlated, differences in individual day 0 F1 due to parasitaemia at enrolment were explored, i.e. baseline parasite counts recorded at the first treatment day, by combining these two covariates. Furthermore, it was hypothesized that a patient’s clinical condition affects MQ K_a_ and this was tested by integrating the effect of the treatment day (0 vs. 1 and 2) on K_a_.

Terminal half-lives (t_1/2_), maximum concentration (C_max_), and time to achieve C_max_ (t_max_) for all the three drugs, MQ area under the curve to infinite (AUC_0–inf_) and AS and DHA AUC_0–24_ after the first and the third ASMQ intake were computed using final pharmacokinetic parameter estimates and classic pharmacokinetic equations or NONMEM integration, as appropriate.

### Parameter estimation, model selection and exclusion criteria

MQ and AS/DHA concentrations were fitted using the first-order conditional (FOCE) method with interaction. AS and DHA non-zero concentrations measured more than a week after last drug intake were thought unreliable and thus omitted from the analysis. Other missing variables (unreported concentration measurements, dose intake or sampling times, inconsistent date/time of dose intake and sampling) were also omitted. Data below the quantification limit (BQL) of the assays were handled by setting the first of a series of BQL samples at LOQ/2 and as missing all the others (M6 method) [23].

Diagnostic goodness-of-fit plots, along with differences in the NONMEM^®^ objective function value (ΔOFV), were employed to discriminate between nested models. Since a ΔOFV between any two hierarchical models approximates a χ^2^ distribution, a change of more than 3.84 (p < 0.05) and 6.63 (p < 0.01) points was considered statistically significant for one additional parameter in model-building or forward insertion and backward-deletion covariate steps, respectively. Akaike’s information criterion (AIC) was used for non-hierarchical models. Shrinkage was also evaluated. Sensitivity analyses removing outlying data with absolute conditional weighted residuals (CWRES) greater than 4 or potentially unreliable covariate values and concentration measurements were finally performed to avoid any potential bias in parameter estimation and covariate exploration.

#### Model validation and assessment

The stability of the final MQ and AS/DHA models was assessed by means of the bootstrap method implemented in PsN-Toolkit [15]. Median parameter values with their 95% confidence interval (CI_95%_) were derived from 2000 replicates of the initial datasets and compared with the original estimates. Prediction-corrected visual predictive checks (pcVPC) were also performed using the PsN-Toolkit and the R package Xpose4 by simulations based on the final pharmacokinetic models with variability using 1000 children [15, 24]. Moreover, the final MQ pharmacokinetic model was validated using concentrations collected from participants not used in initial model development. The accuracy and precision of the model were estimated by means of prediction error (MPE) and root mean square error (RMSE), using log-transformed concentrations, for the entire dataset and also for each study site [25].

### Comparison between mefloquine exposures in children and adult volunteers and patients

Median and 90% prediction interval (PI_90 %_) of children and adult concentration–time profiles were obtained through simulations (n = 1000) using the final pharmacokinetic model described above and published MQ pharmacokinetic models including BSV and intra-individual variability, respectively. A literature search allowed the identification of two pharmacokinetic models developed in adults receiving the same fixed dose formulation of ASMQ as the one administered to the children enrolled in this clinical trial [26, 27]. The investigated populations consisted of Indian adult patients and Thai adult patients and volunteers, administered with 400 mg of MQ once per day over three consecutive days. MQ disposition was described by a two compartment model with linear elimination in both analyses. A first-order and a single transit compartment models in Julien et al. [26] and Reuter et al. [27], respectively, characterized the absorption phase. The two models were implemented in NONMEM^®^, fixing simulated individuals’ body weight to the corresponding median population value. Administered MQ doses were 110 mg and 400 mg over three consecutive days for children and adults, respectively. MQ drug exposure was quantified by computing median and PI_95%_ AUC over the whole study period (AUC_0–day63_) by NONMEM integration for all the simulated population/model.

#### Mefloquine pharmacokinetic–pharmacodynamic analysis

This exploratory analysis was carried out on MQ data collected from all children participating in the trial with complete dosing history information that did not drop out in the early days of the study. Model predicted MQ cumulative AUC (AUC_0–dayx_) on study days 7, 28, 42, and 63 were calculated by concentration integration in NONMEM^®^. The relationship between recrudescence of infection (response variable, coded as 0/1) and model predicted AUC_0–dayx_ (independent variable) on study days 7, 28, 42, and 63 was inspected by means of logistic regression using STATA (StataCorp. 2013. Stata Statistical Software: Release 13. College Station, TX: StataCorp LP). The independent variable was log-transformed (using base 2) and cantered on its median value. The level of significance was set at 0.05.

## Results

Of the 472 children enrolled in the trial and randomized in the ASMQ arm, 21 were removed according to the exclusion criteria of the pharmacokinetic analysis. MQ and AS/DHA pharmacokinetic model development was carried out on 48 patients and MQ model validation on 378 patients, after removal of subjects with unreliable data. The characteristics of the patients used in the MQ and AS/DHA model-building, as well as the final MQ model validation and MQ pharmacokinetic–pharmacodynamic analysis datasets, are listed in Table 1.

**Table 1:** Characteristics of the children enrolled in the trial for mefloquine and artesunate/dihydroartemisinin model development, mefloquine model validation and pharmacokinetic-pharmacodynamic analysis

Baseline characteristic	Model-building dataset (n = 48)		MQ validation dataset (n = 378)		MQ pharmacokinetic–pharmacodynamic analysis dataset (n = 451)	
	Value	% or range	Value	% or range	Value	% or range
Demographic characteristics						
Sex (male/female) (no.)	19/29	40/60	183/195	48/52	219/232	49/51
Median age (year)	2.6	0.6–5.0	2.3	0.5–5.0	2.4	0.5–5.0
Median body weight (kg)	12	7–17	11	5–18	11	5–19
Median height/length (cm)	89	66–114	85	62–114	86	60–114
Physiological characteristics						
Total bilirubin (μmol/L)	10	1–77	10	0.4–74	17	0.5–163
Serum creatinine (μmol/L)	42	3–119	36	2–86	40	3–120
Haematocrit (%)	31	17–49	30	16–59	30	17–62
Haemoglobin (g/dL)	10	5–15	9	5–18	9	5–19
AST (IU)	39	5–302	34	7–576	39	5–680
ALT (IU)	19	7–449	16	4–378	18	3–814
Baseline parasite counts (no/μL)						
Median at day 0	58,131	3322–190,896	–	–	–	–
Median at day 1	280	0–13,816	–	–	–	–
Median at day 2	0	0–100	–	–	–	–
Co-administered drugs						
CYP3A4 inducers (at least for one drug measurement/never) (no.)	7/41	15/85	–	–	–	–

AST, aspartate aminotransferase; ALT, alanine aminotransferase

### Population pharmacokinetic analysis

A total of 216 MQ, 117 AS and 134 DHA (including BQL) concentrations were available for the 48 Kenyan patients selected for the pharmacokinetic model development. Of note, none of the MQ concentrations were quantified as a BQL, while 71% and 57% of AS and DHA samples were BQL data. Median (range) treatment duration per study subject was 3 days (1–3) and the number of available non-BQL samples was 5 (1–7) for MQ, 1 (1–2) for AS and 2 (1–3) for DHA. MQ concentrations ranged between 0.17 ng/mL and 6552.51 ng/mL, AS (> BQL) between 2.1 and 8469.8 ng/mL and DHA (> BQL) between 2.9 and 2400.9 ng/mL.

### Artesunate and dihydroartemisinin

#### Structural and statistical model

As previously described, a two-compartment model was used to simultaneously fit AS and DHA data with first-order absorption, drug exclusive elimination via irreversible conversion to DHA and first-order elimination of metabolite. Initially, BSV was assigned only on CL and a mixed error model was assumed for the intra-patient variability of both drug and metabolite. Model stability was achieved by integrating a correlation between AS and DHA concentration measurements (ΔOFV = − 25, p < 0.001). BSV on V_C_ did not improve data description (ΔOFV = 0, p > 0.05) whilst assignment of BSV to CL_M_ (ΔOFV = − 7.3, p < 0.01) and to V_M_ (ΔOFV = − 8.0, p < 0.01) yielded a better fit of the data. Inclusion of relative F1 (fixed to 100% with estimated BSV) explained all the BSV on AS and DHA clearance and significant decreased the OFV (ΔOFV = − 17.7, p < 0.01). The estimates and variability (CV%) of the pharmacokinetic parameters obtained by the base population model were a relative F1 of 100% (67%), a CL of 180 L/h, a V_C_ of 166 L, a CL_M_ of 12.5 L/h and a V_M_ of 13.8 L (57%).

#### Covariate analysis

Age, sex and BIL as well as the hepatic liver tests ALT and AST had a significant impact on F1 (ΔOFV < − 9.6, p < 0.01). Because of poor effect estimation (relative standard error, RSE = 155%), BIL was not kept for further covariate analyses. Sensitivity analyses revealed that the effect of ALT and AST on F1 were purely due to a single patient having the highest values for both hepatic enzyme tests. Whether this finding was a true or an incidental effect could not be validated and these covariates were thus not retained in the model. F1 was found to increase with the parasite counts (ΔOFV = − 13.2, p < 0.01), and to be higher at day 0 compared to days 1 and 2 of treatment (ΔOFV = − 13.7, p < 0.01). As shown in Table 1, baseline parasite counts were extremely high before starting the anti-malarial treatment and dropped to 0 before administration of the third ASMQ, a consequence of the important and immediate AS effect. Differences in F1 at day 0 related to parasite counts were investigated but did not improve the fit with respect to the model including only the treatment day or the parasite counts as covariate (ΔOFV < 3.8, p > 0.05). Because of the correlation between the two factors and the absence of fit improvement by combining the parasite information and the treatment day, only the latter was kept in the model. BW allometric scaling on CL_M_ and V_M_ markedly decreased the objective function (AIC difference of − 22 with respect to the basic structural model). Maturation on CLM was adequately described using Eq. 3 and improved the model fit (ΔOFV = − 18.9, p < 0.01). V_M_ was significantly impacted by sex (ΔOFV = − 8.8, p < 0.01). Complete multivariate analyses allowed for the effect of sex on V_M_ and F1 to be discarded, as well as that of maturation on DHA clearance. These results show that F1 is reduced by 68% upon doubling child age with respect to the population median (2.6 years), and is 29% higher in the first day of therapy than in the subsequent treatment days. The effect of BW on CL_M_ and V_M_ was also retained.

#### Model evaluation and assessment

**Fig. 1:**
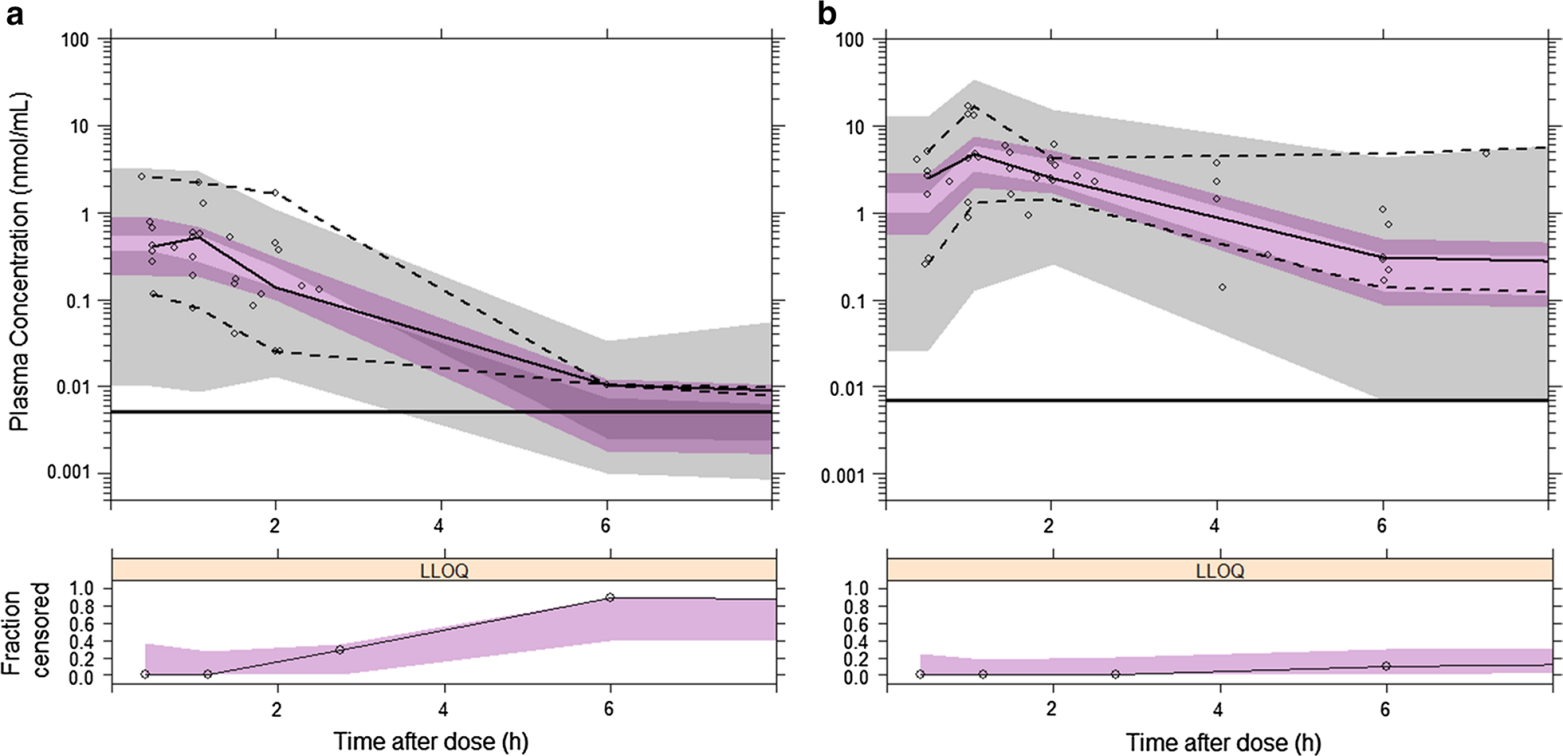
Prediction corrected visual predictive check of the final model of **a** artesunate and **b** dihydroartemisinin. Open circles represent prediction corrected observed plasma concentration; black solid and dashed lines the median and PI_90%_ of the observed data; shaded magenta and grey surfaces the model predicted 90% confidence interval of the simulated median and PI_90%_, respectively; horizontal black lines are the LOQ of AS (0.005 nmol/mL) and DHA (0.005 nmol/mL). The lower panels show the fraction of observed (open circles) with the PI_95%_ of the simulated (shaded magenta surface) BQL data

The final model parameter estimates, together with their bootstrap estimations, are shown in Table 2 and the goodness-of-fit plots in Additional file 1. Model predicted secondary parameters are presented in Table 4. Shrinkage was lower than 30% for BSV and 10% for residual variabilities. The model was considered reliable since the parameter estimates were within the bootstrap CI_95%_ and differed less than 15% from their bootstrap estimations. Prediction corrected VPCs shown in fig. 1 evidence model misspecification. However, the model was judged acceptable because of the paucity of available AS/DHA data.

**Table 2:** Final population parameter estimates of artesunate and dihydroartemisinin with their bootstrap evaluations in 2000 replicates

Population pharmacokinetics analysis					Bootstrap evaluation			
Parameter	Estimate	RSE (%)^a^	BSV (%)^b^	RSE (%)^a^	Estimate	CI_95%_^c^	BSV (%)^b^	CI_95%_^c^
F1 (%)	100 fixed		56	16	100 fixed		53	29 to 70
θ_day F1_	− 0.29	54			− 0.33	− 0.57 to 0.05		
θ_age F1_	− 0.68	19			− 0.68	− 0.91 to − 0.31		
CL (L/h)	146	20			139	91 to 202		
V_C_ (L)	139	23			131	78 to 199		
K_a_ (h^−1^)	3.2 fixed				3.2 fixed			
CL_M_^d^ (L/h)	11	15			11	8 to 15		
V_M_^d^ (L)	11	20	60	29	10	7 to 15	49	16 to 75
σ_prop,AS_ (CV%)	79	8			78	65 to 92		
σ_prop,DHA_ (CV%)	60	9			60	48 to 69		
Corr_prop_ (%)	44	36			43	40 to 64		
σ_add,AS_ (nmol/mL)	0.0023	3			0.0023	0.0022 to 0.0025		
σ_add,DHA_ (nmol/mL)	0.0042	13			0.0041	0.0035 to 0.0049		

F1, AS relative bioavailability; CL, AS clearance; V_C_, AS central volume of distribution; K_a_, first-order absorption rate constant; CL_M_, DHA clearance; V_M_, DHA volume of distribution; σ_prop_, exponential residual error; σ_add_, additive residual error; corr_prop_, correlation between the proportional error components; θ_age F1_, age effect on F1 expressed as (1 + θ_age F1_(AGE-MAGE)/MAGE) with MAGE = 2.6 years; median AGE value in the study population; θ_day F1_, day effect on F1 expressed as (1 + θ_day F1_Q1) with Q1 = 0 for the first treatment day; 1 for subsequent therapy days

^a^Relative standard error (RSE) of the estimate defined as SE estimate/estimate, expressed as a percentage, with SE estimate retrieved directly from the NONMEM output file

^b^Between-subject variability

^c^95% confidence interval (CI)

^d^Pharmacokinetic parameter of a patient of 12.2 kg, the median population body weight (MBW). DHA individual clearance, and volume of distribution are obtained by the equations: CL_M,ind_ = CLM*(BW/MBW)^0.75^ and V_M,ind_ = V_M_*BW/MBW, respectively, with BW patient’s body weight

### Mefloquine

#### Structural and statistical model

A two-compartment model with first-order absorption and elimination described MQ data better than a one-compartment model (ΔOFV = − 64, p < 0.001). No additional benefit was observed using three compartments (ΔOFV = − 0.9, p > 0.05). BSV on V_C_ (ΔOFV = − 22, p < 0.001) in addition to CL yielded a better fit of the data, which was further enhanced by inclusion of BSV on K_a_ (ΔOFV = − 19, p < 0.001). No improvement of the model fit was observed associating BSV on Q or V_P_ (ΔOFV = 0, p > 0.05). The inclusion of MQ F1 fixed to 100% with an estimated BSV significantly decreased the OFV whilst explaining all the BSV associated to V_C_ (ΔOFV = − 9.4, p < 0.01). Finally, a proportional model was retained to describe the intra-patient variability. The estimates and variability (CV %) of the pharmacokinetic parameters obtained by the base population model were an F1 of 100% (39%), a CL of 0.48 L/h (40%), a V_C_ of 88 L, a Q of 0.41 L/h, a V_P_ of 69 L, and a K_a_ of 0.15 h^−1^ (87%).

#### Covariate analysis

The univariate analyses showed no association between the covariates tested and MQ bioavailability, clearances and volumes of distribution (ΔOFV ≥ − 3.2, p > 0.05; AIC difference of 2 points with respect to the structural model for BW on all the PK parameters). However, the sensitivity analysis performed while removing the patient with extremely low concentrations after the second and third ASMQ dose revealed that this outlier masked the real impact of BW on clearances and volumes of distribution and of age on F1 (AIC difference of − 5 and ΔOFV = − 5.4, p < 0.05, respectively), without inducing any modification in the MQ basic model. Sex and age were found to significantly influence K_a_ (ΔOFV ≤ − 7.0, p < 0.05). A decrease of 74% in K_a_ was observed while doubling the age with respect to the population median (2.6 year) and female children had 55% lower K_a_ than male children. Multivariate analysis showed that age accounted for the effect of sex on K_a_ and allowed for the discarding of the impact of age on F1. Finally, significantly different K_a_ at day 0 and 1/2 of ASMQ treatment were identified due to improvement in patient health following the first intake of AS (ΔOFV = − 39.2, p < 0.001). Multivariate and backward deletion step analyses performed using the reduced dataset, obtained by removal of the outlying patient, showed that the BW effect on clearances and volumes of distribution, as well as age and treatment day effect on K_a_, should be retained in the final MQ pharmacokinetic model.

#### Model evaluation and assessment

**Fig. 2:**
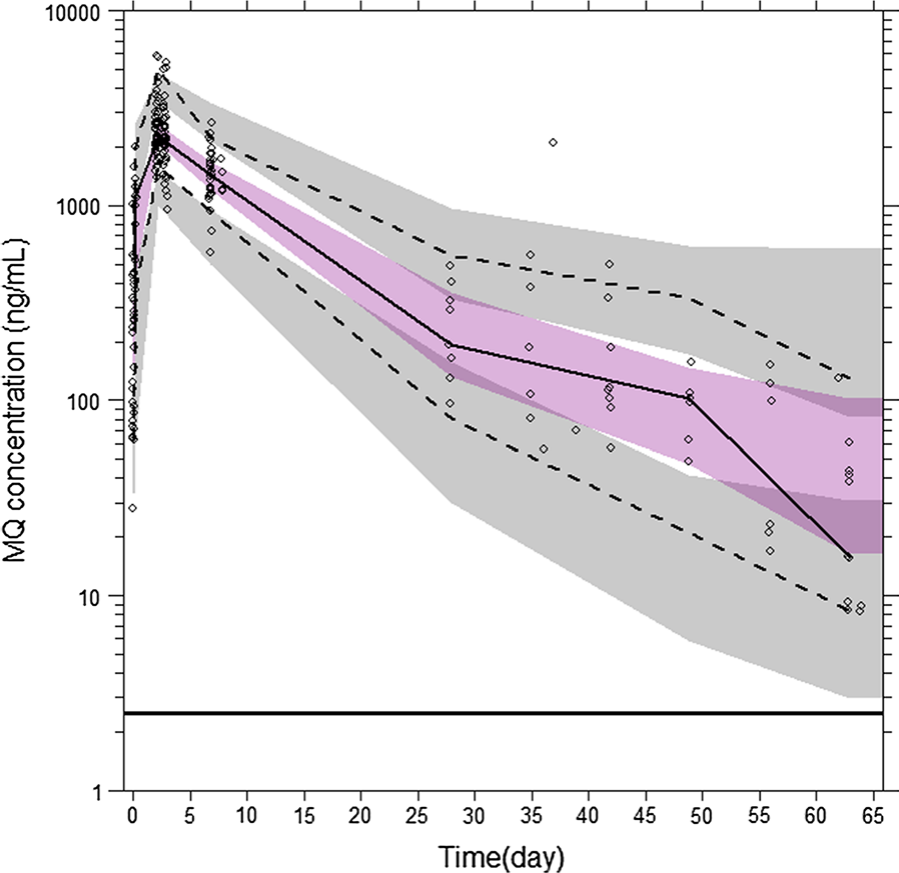
Prediction corrected visual predictive check of the final model with MQ prediction corrected plasma concentration (open circles) and quartiles (black solid and dashed lines) with model-based percentiles 90% confidence interval (shaded magenta and grey surfaces for the median and low/high percentiles, respectively). Horizontal black line represents the MQ LOQ (2.5 ng/mL)

The final model parameters, together with their bootstrap estimations, are displayed in Table 3 and the goodness-of-fit plots presented in Additional file 2. Model predicted secondary parameters are shown in Table 4. Shrinkage was 28% for residual variability and lower than 15% for BSV. The model was considered reliable since the parameters were within the bootstrap CI_95%_ and differed less than 5% from the bootstrap estimations. The results of the pcVPC (Fig. 2) support the predictive performance of the model. Moreover, the external validation done using the remaining 538 concentrations from 378 children enrolled in the trial showed a negligible bias of 0% (CI_95%_ − 2 to 1%) with a precision of 16% at an individual level. A small bias of 18% (CI_95%_ 13–24%) with a precision of 81% was calculated for population predictions. Non-significant or small (absolute values ≤ 6%) biases were calculated at each study site on an individual level (Table 5). Furthermore, the precision of drug predictions was close to the estimated residual intra-patient variability, which strongly supports the predictive performance of the model (Table 5).

**Table 3:** Final population parameter estimates of mefloquine with their bootstrap evaluations in 2000 replicates

Population pharmacokinetics analysis					Bootstrap evaluation			
Parameter	Estimate	RSE (%)^a^	BSV (%)^b^	RSE (%)^a^	Estimate	CI_95%_^c^	BSV (%)^b^	CI_95%_^c^
F1	1 FIX		28	15	1 FIX		27	18 to 34
CL (L/h)^d^	0.45	7	39	17	0.45	0.39 to 0.51	38	24 to 51
V_C_ (L)^d^	95	7			92	66 to 106		
K_a_ (h^−1^) DAY = 1	0.17	17	91	12	0.16	0.10 to 0.23	87	64 to 110
K_a_ (h^−1^) DAY > 1	0.40	22			0.38	0.22 to 0.64		
θ_AGE Ka_	− 0.67	18			− 0.66	− 0.91 to − 0.29		
Q (L/h)^d^	0.35	28			0.35	0.24 to 1.30		
V_P_(L)^d^	60	9			61	51 to 82		
σ_prop_ (CV %)	21	13			20	14 to 26		

F1, bioavailability; CL, clearance; V_C_, central volume of distribution; K_a_, first-order absorption rate constant; Q, intercompartmental clearance; V_P_, peripheral volume of distribution; σ_prop_, exponential residual error; θ_AGE Ka_, effect of AGE on K_a_ expressed as (1 + θ_AGE Ka_ (AGE–AGE)/MAGE) with MAGE = 2.6 years; median AGE value in the study population

^a^Relative standard error (RSE) of the estimate defined as SE estimate/estimate, expressed as a percentage, with SE estimate retrieved directly from the NONMEM output file

^b^Between-subject variability

^c^95% confidence interval (CI)

^d^Pharmacokinetic parameter of a patient of 12.2 kg, the median population body weight (MBW). Individual clearance, peripheral clearance and volumes of distribution are obtained by the equations: CL_M,ind_ = CL*(BW/MBW)^0.75^, Q_M,ind_ = Q*(BW/MBW)^0.75^, V_C,ind_ = V_C_ * BW/MBW, and V_P,ind_ = V_P_ * BW/MBW, respectively, with BW patient’s body weight

**Table 4:** AS, DHA and MQ final model-predicted secondary pharmacokinetic parameters

Derived parameter [median (PI_95%_)]	AS	DHA	MQ
C_max_	0.52 (0.17, 1.43) nmol/mL	3.9 (1.0, 11.4) nmol/mL	2874 (1099–4994) ng/mL^a^
t_max_ (h)	0.52	1.4 (1.0–1.7)	56 (35–62)^a^
t_1/2_	40 min	40 (20–81) min	12 (9–24) day
AUC_0–24, day 0_ (ng/L/h)	0.34 (0.12, 0.93)	3.30 (0.88, 9.30)	–
AUC_0–24, day 2_ (ng/L/h)	0.23 (0.08, 0.64)	2.20 (0.60, 6.30)	–
AUC_0–inf_ (ng/L/h)	–	–	650 (251–1619)

C_max_, maximum concentration; t_max_, time to achieve C_max_; t_1/2_, terminal half-life; AUC_0–24, day0_ and AUC_0–24, day2_, area under the curve (area under the curve) after the first and third ASMQ intake, respectively; AUC_0–inf_, AUC to infinite

^a^Two patients received only 1 MQ dose and have a t_max_ < 50 h with a C_max_ < 1000 ng/mL

**Table 5:** Final model accuracy and precision per study site at individual level

Study sites	Observations (patients)	MPE (CI_95%_) (%)	RMSE (%)
Tanzania site no 1	24 (18)	− 5 (− 9 to − 1)	12
Kenya site no 2	188 (123)	0 (− 2 to 2)	14
Burkina Faso site no 3	158 (110)	0 (− 2 to 2)	13
Burkina Faso site no 4	78 (55)	2 (− 4 to 8)	30
Tanzania site no 5	68 (52)	0 (− 4 to 2)	14
Tanzania site no 6	22 (20)	− 4 (− 7 to − 2)	9

MPE, mean prediction error, calculated as: exp(mean(ln(IPRED/DV)))−1; RMSE, root mean square error, calculated as exp(sqrt(mean(ln(IPRED/DV))^2^))−1. IPRED and DV represent the individual predicted and observed concentration, respectively

### Comparison between mefloquine exposures in children and adult volunteers and patients

Horizontal black line represents the MQ LOQ (2.5 ng/mL).

**Fig. 3:**
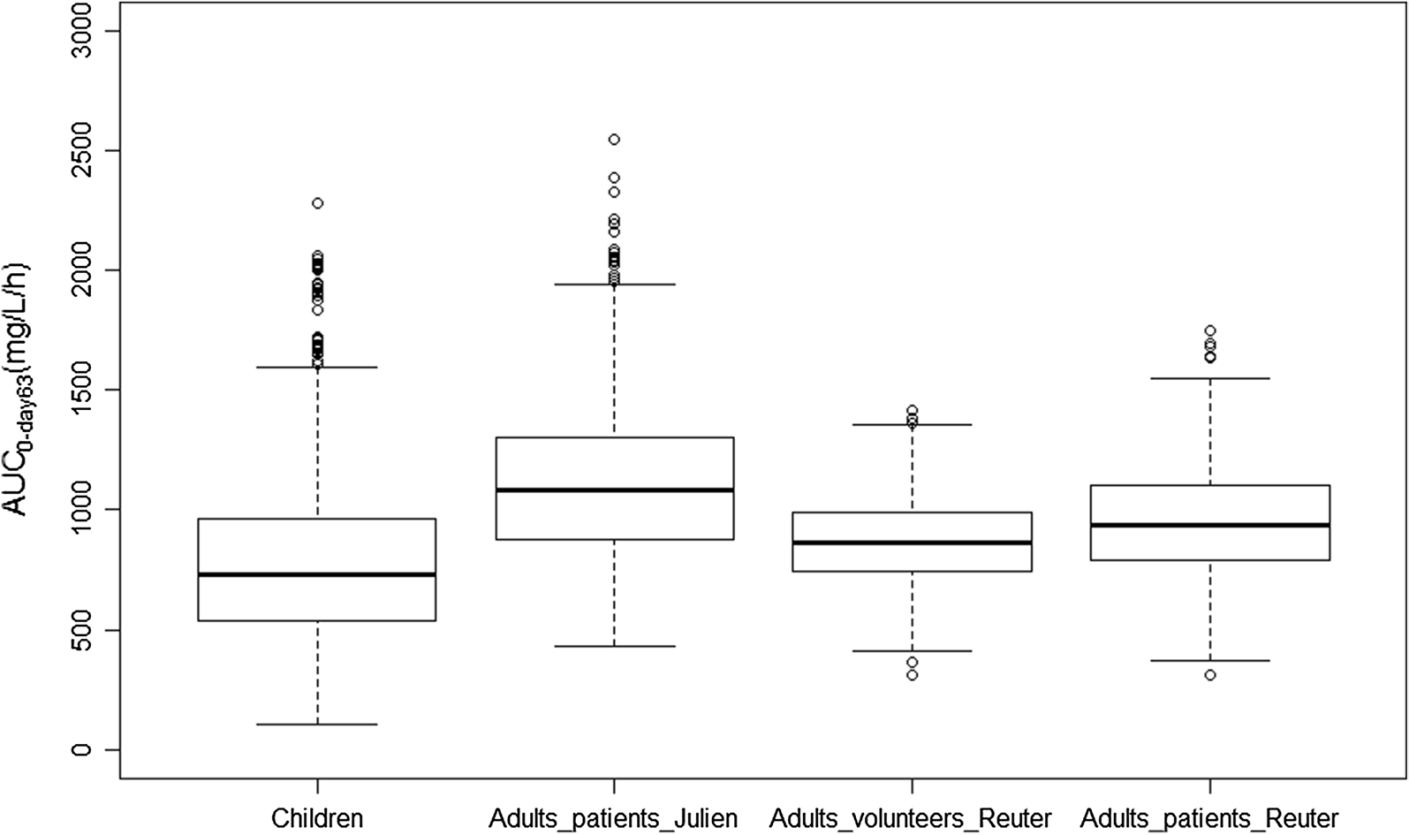
Model predicted AUC_0–day63_ for children and adult patients and volunteers obtained by simulating 1000 individuals with the present (children), the Julien et al. (adult patients) and Reuter et al. (adult volunteers and patients) models, respectively [26, 27]

**Fig. 4:**
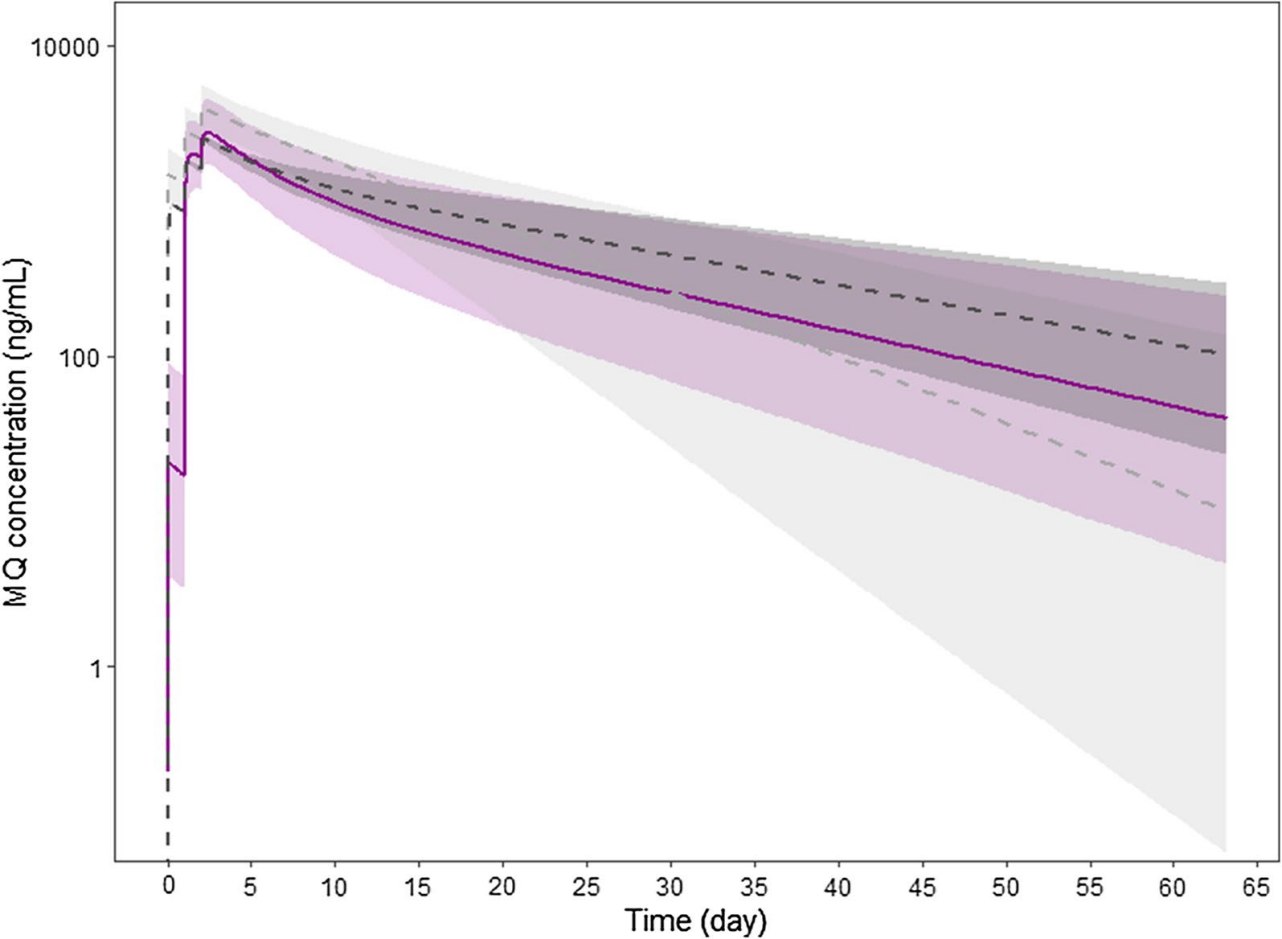
Median and 90% prediction intervals of MQ concentration–time profiles for children and adult patients receiving 110 mg and 400 mg of MQ once per day over three consecutive days obtained with this study (children, magenta solid line and shaded surface), the Julien et al. (adult, light grey line and shaded surface), and Reuter et al. (adult, dark grey line and shaded surface) models, respectively [26, 27]

Figure 3 compares the model-predicted AUC_0–day63_ for children and adult volunteers and patients. Median (PI_95%_) AUC_0–day63_ of 725 mg/L/h (310–1718) was computed through simulations of the final pharmacokinetic model for children weighting 12.2 kg and taking 110 mg of MQ once per day over three consecutive days. Adult patients had a median (PI_95%_) AUC_0–day63_ of 1080 mg/L/h (599–1911) and 936 mg/L/h (570–1413) calculated using the model of Julien et al. and Reuter et al. respectively, while adult volunteers of 865 mg/L/h (555–1211) under the dosage regimen of MQ 400 mg once per day over three consecutive days. Median (PI_90%_) concentration time profiles for adult and children patient are shown in Fig. 4.

### Mefloquine pharmacokinetic-pharmacodynamic analysis

Treatment failure was reported for 212 (56%) of the children enrolled in the study, of these failures, 81% (n = 171) were due to new infections and 7% (15) to recrudescence during the 63 days of follow-up. In 2% of the enrolled individuals PCR information was missing and in 10% it was not possible to determine the nature of the treatment failure. Median (range) model-predicted AUC_0–day7_ were estimated to be 281 mg/L/h (70–854 mg/L/h) in children with reported treatment success within the follow-up period, and 286 mg/L/h (167–378 mg/L/h) and 286 mg/L/h (70–579 mg/L/h) for children with or without malaria recrudescence, respectively. No significant associations were found through logistic regression between model-predicted AUC_0–day_ at day 7, 28, 42 or 63 and appearance/absence of recrudescence (p > 0.05) (data not shown for day > 7).

## Discussion

The present analysis describes the pharmacokinetics of fixed-dose ASMQ in African children under the age of 5 years, with the aim of identifying the source of the significant variability in drug exposure and validating the recommended weight-for-age dosage regimen. The very short half-lives estimated for AS and DHA are in good agreement with reported values ranging from 22 to 72 min for the drug and from 30 to 186 min for the metabolite [28, 29]. The calculated t_max_ for AS and DHA agree with reported peak AS and DHA plasma concentrations within the 1st h and 2 h post-dose, also supporting the appropriateness of the value chosen for AS K_a_ in this work [28, 29].

A two-compartment model was used to describe mefloquine pharmacokinetics as already shown in previous analyses [26, 27]. Drug clearance and central and peripheral volumes of distribution were found to be markedly lower than the values estimated in adult patients of African, Caucasian or Asian origin [26, 27, 30]. However, estimated median terminal half-life and mean absorption times are comparable to those obtained for adults [26, 27, 30–32]. In addition, simulations performed using the final model in children and previously published pharmacokinetic models in adult patients and volunteers show that these populations have comparable exposure under the specific recommended dosing regimen.

Considerable between-subject variability characterized the pharmacokinetics of both anti-malarial drugs. Such variability remained largely unexplained by the inclusion of the available covariates. Body weight was associated to all the MQ and DHA pharmacokinetic parameters. The association between this demographic characteristic and AS and DHA dispositions has already been described [17, 33]. Reported discrepancies in MQ disposition and elimination between adult and children may be ascribed to differences in patients’ body weight. These results illustrate the association between body weight and AS/DHA and MQ dispositions after ASMQ FDC administration in African children and thus support the recommended dose adjustments according to weight-for-age, in order to obtain similar exposures in adults and children.

Twenty-one percent of the initial AS relative F1 variability was explained by age and treatment day. It is worth realizing that F1 is intrinsically connected to AS and DHA pharmacokinetic parameters, apparent because of ASMQ oral administration. The decrease in F1 observed with age implies an increase in drug and metabolite eliminations. This effect might thus be related to organ maturation in the study population. F1 was significantly higher at day 0 than days 1 and 2 of treatment. This is a consequence of the rapid and efficacious therapeutic AS effect observed already after the first AS dose intake. Recently, the relationship between malaria disease and AS bioavailability has been described using parasitaemia variation [34, 35]. An increase of AS F1, resulting in augmented drug exposure, was reported with increasing parasite counts. This same trend was found in univariate analysis in the study population but was not retained after multivariate combination with treatment day. These two covariates are indeed correlated. However, it was not possible to identify differences in the first dose F1 due to variations in parasite counts in the study population. This suggests that the general health improvement and not only the disappearance of the parasite after the first ASMQ dose affects AS pharmacokinetics.

Age was found to markedly decrease MQ drug absorption rate. A significantly higher t_max_ has been reported in healthy fasting volunteers taking an MQ dose compared to those having a high-fat breakfast (36 vs. 17 h), meaning that food would increase MQ K_a_ [36]. This is consistent with the hypothesis that the younger children in the African paediatric population investigated were breastfed and thus received a more appropriate amount of food compared to the older ones. Under this hypothesis, and according to the results of the previously cited study, younger children are expected to have higher MQ K_a_ than older ones. Of note, the impact of food on MQ K_a_ remains controversial [37, 38]. Finally, the rapid and significant therapeutic AS effect, captured in the analysis by treatment day, induced a significant increase in MQ absorption rate after the first ASMQ FDC administration. It is possible that the dramatic decrease of parasite load following the first intake of AS improves patient state of health resulting in the disappearance of gastrointestinal tract disturbances [22]. The PK of the second and third MQ doses thus might benefit from the AS treatment effect with a favourable modification of drug absorption rate.

As already described in studies performed in Tanzania and Cambodia, more than half of the African paediatric participants had a residual concentration of at least one anti-malarial drug above the limit of quantification at baseline (lumefantrine was measured in 64% of the patients, sulphadoxine in 11%, amodiaquine/deshethylamodiaquine in 16%, pyrimethamine in 2% and quinine in 6%) [39, 40]. These findings are worrying since they indicate that parasites have been exposed to sub-therapeutic concentrations of anti-malarials for some time in a population presenting an elevated risk of developing drug resistance [22]. This might contribute to the dangerous spread of anti-malarial drug resistance.

The MQ model developed in Kenyan children using intensive sampling was applied to data collected from children from Burkina Faso, Tanzania and Kenya. Similar non-significant or small biases and precision per study centre were estimated, suggesting comparable drug exposure among different populations. The relationships between therapeutic response and pharmacokinetics of MQ as monotherapy and in combination with AS have been previously compared in a Thai population [41]. Recrudescence of infection in 24% and 2% of patients was reported in cases of MQ administered alone and with AS, respectively, indicating that the addition of the artemisinin derivative improved the cure rates considerably. Furthermore, no significant association could be found between MQ pharmacokinetics and treatment response. In line with these results, only 3% of the African paediatric population studied presented recrudescence of infection, which could not be related to MQ exposure within the study period. The low number of cases of malaria recrudescence might have limited the likelihood of detecting such an association.

## Conclusions

The study described provides the pharmacokinetic parameters for MQ and AS, administered as a FDC of AS/MQ, in African children under the age of 5 years with acute *P. falciparum* malaria. The considerable variability characterizing the pharmacokinetics of these two anti-malarial drugs can be partly explained by children’s body weight, justifying the current dosing recommendations based on weight-for-age considerations, to ensure similar exposure in children and adults.

## Additional files

**Additional file 1.** Artesunate (upper panel) and dihydroartemisinin (lower panel) goodness-of-fit plots of observed vs. individual and population predicted concentrations, and conditional weighted residuals (CWRES) vs. population predicted concentrations and time after dose.

**Additional file 2.** Mefloquine goodness-of-fit plots of observed vs. individual and population predicted concentrations, and conditional weighted residuals (CWRES) vs. population predicted concentrations and time after dose.

## Abbreviations

ACT: artemisinin-based combination therapy
AIC: Akaike’s information criterion
ALT: alanine aminotransferases
AMLF: artemether–lumefantrine
ASMQ: artesunate–mefloquine
AST: aspartate
AUC: area under the curve
BIL: total bilirubin
BQL: below the quantification limit
BSV: between-subject variability
BW: body weight
CL: clearance (CL for drugs and CL_M_ for metabolite)
C_max_: maximum concentration
CWRES: conditional weighted residuals
DHA: dihydroartemisinin
DND*i*: Drugs for Neglected Diseases *initiative*
ESI: electrospray ionization
F1: relative bioavailability
FACT: fixed-dose artesunate-based combination therapy consortium
FDC: fixed dose combination
FOCE: first-order conditional method
Hb: haemoglobin
Ht: haematocrit
K_a_: absorption rate constant
K_mat_: the age maturation rate constant
LOQ: limits of quantification
MAT_mag_: maturation magnitude for age
MPE: means of prediction error
OFV: objective function value
PAR: pharmacokinetic parameters
PcVPc: prediction-corrected visual predictive checks
PI_90 %_: 90% prediction interval
PK: pharmacokinetics
Q: inter-compartmental clearance
RMSE: root mean square error
t_1/2_: terminal half-life
TM_50_: age at 50% of maturation
t_max_: time to achieve C_max_
TSQ: a triple stage quadrupole
ULN: upper limit of normal
V: central volume of distribution (V_C_ for drugs and V_M_ for metabolite)
V_P_: peripheral volume of distribution
WHO: World Health Organization
WWARN: World Wide Antimalarial Resistance Network
