# Supplementary figures and images for: Population pharmacokinetics and pharmacodynamics of the artesunate–mefloquine fixed dose combination for the treatment of uncomplicated falciparum malaria in African children
Source: Malar J. 2019 Apr 18;18:139. doi: 10.1186/s12936-019-2754-6 (PMC6471806; doi:10.1186/s12936-019-2754-6)

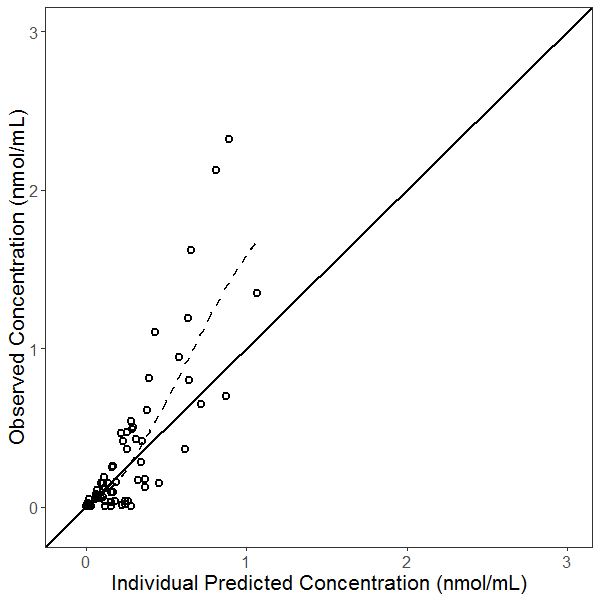

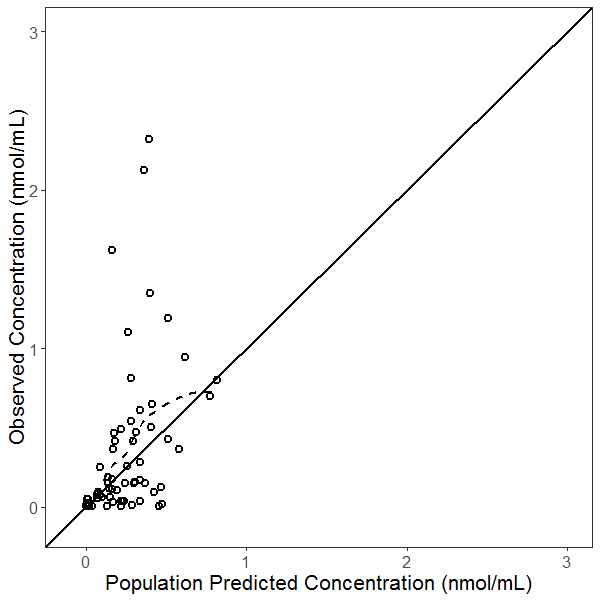


AS


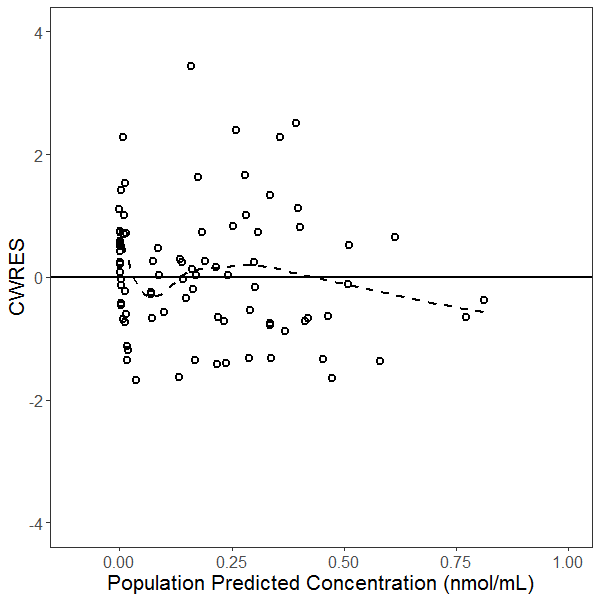

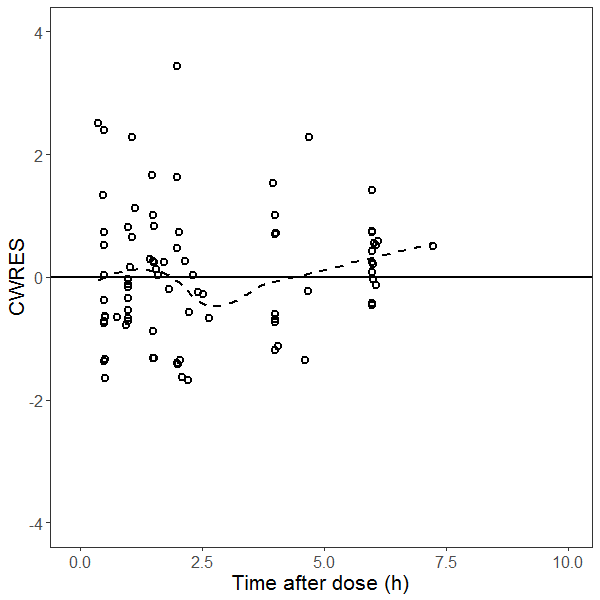


DHA


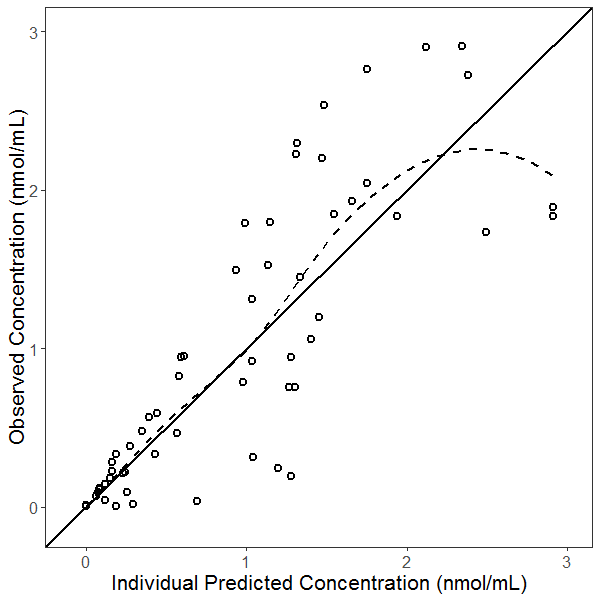

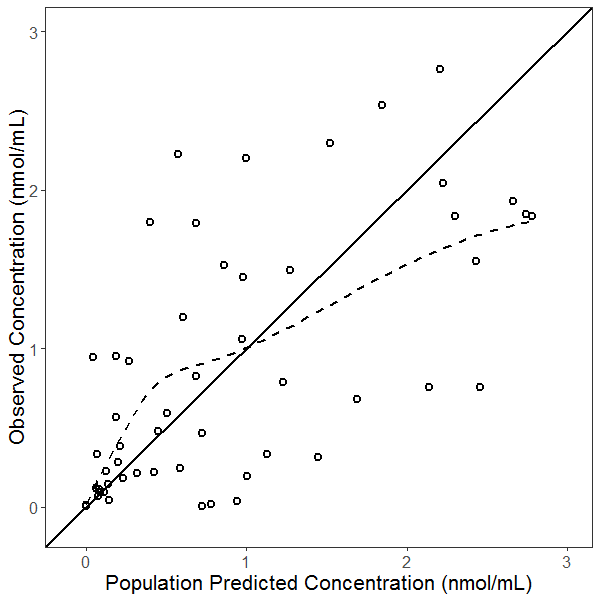


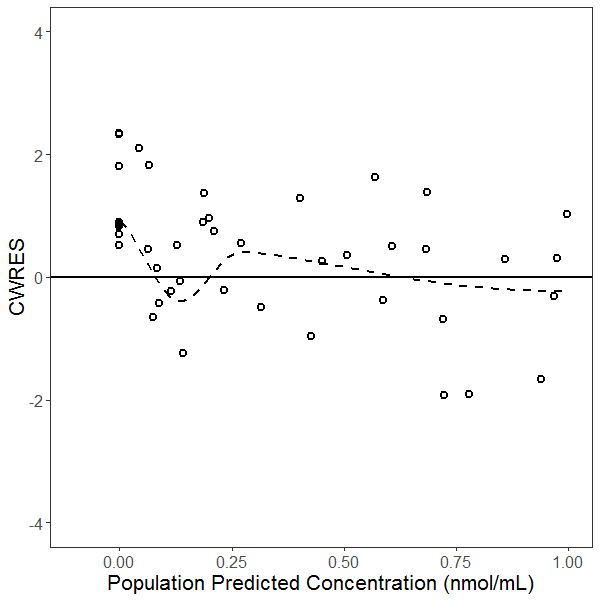

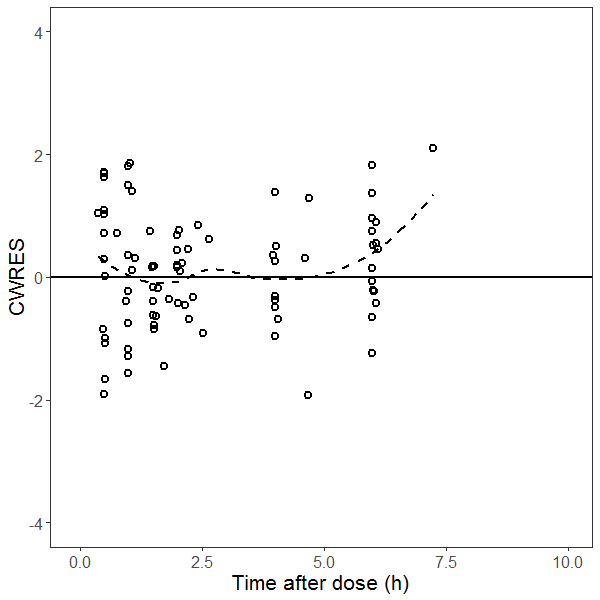

Supplement: Supplementary file 1 — Additional file 1. Artesunate (upper panel) and dihydroartemisinin (lower panel) goodness-of-fit plots of observed vs. individual and population predicted concentrations, and conditional weighted residuals (CWRES) vs. population predicted concentrations and time after dose. [file 12936_2019_2754_MOESM1_ESM.docx]

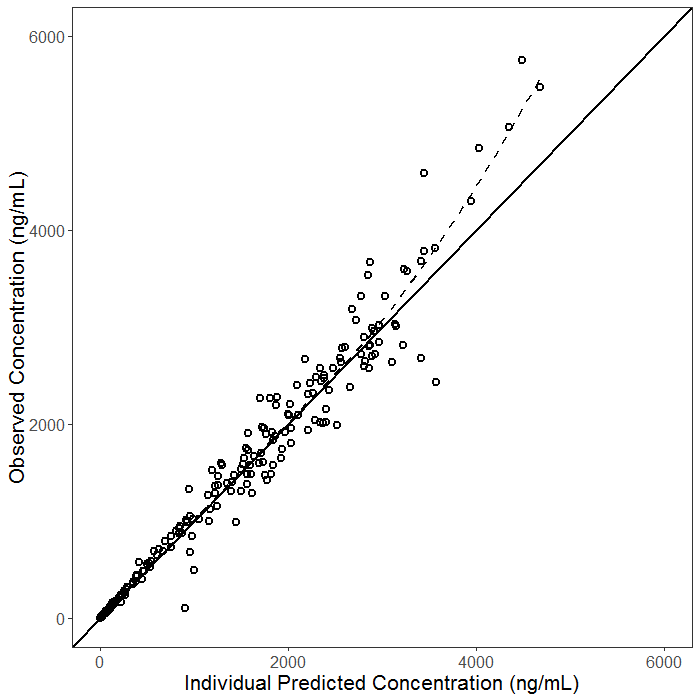

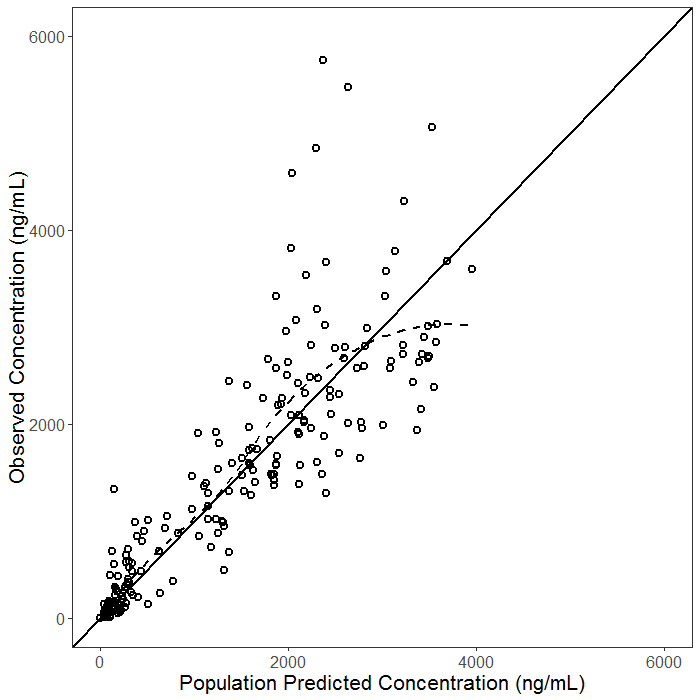

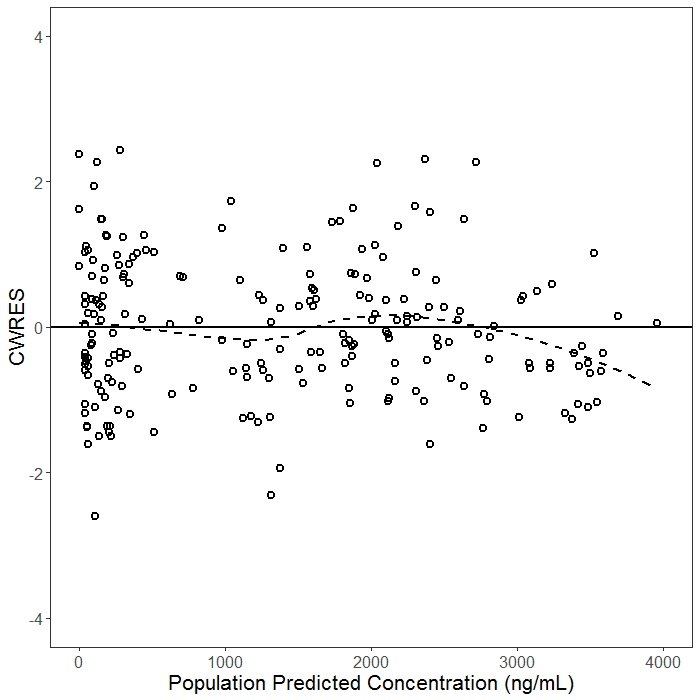

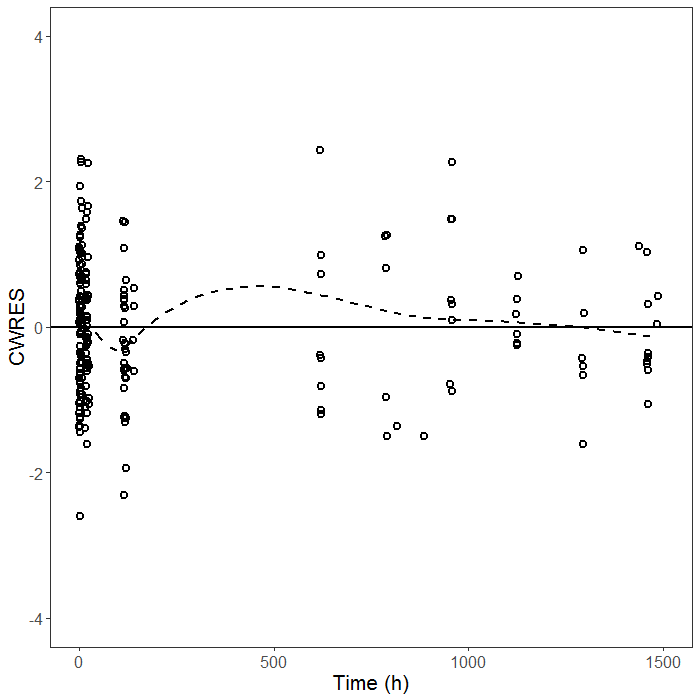

Supplement: Supplementary file 2 — Additional file 2. Mefloquine goodness-of-fit plots of observed vs. individual and population predicted concentrations, and conditional weighted residuals (CWRES) vs. population predicted concentrations and time after dose. [file 12936_2019_2754_MOESM2_ESM.docx]
